# MITO-MODULATORY MEDICATION USE AND SKELETAL MUSCLE BIOENERGETICS IN OLDER ADULTS

**DOI:** 10.1093/geroni/igae098.0428

**Published:** 2024-12-31

**Authors:** Howard Phang, Jaclyn Bergstrom, Rabia Atayee, Laura Hart, Peggy Cawthon, Steve Cummings, Anthony Molina

**Affiliations:** University of California, San Diego, La Jolla, California, United States; UC San Diego, La Jolla, California, United States; University of California San Diego Skaggs School of Pharmacy and Pharmaceutical Sciences, San Diego, California, United States; University of Washington, Seattle, Washington, United States; California Pacific Medical Center, San Francisco, California, United States; California Pacific Medical Center, San Francisco, California, United States; University of California San Diego, La Jolla, California, United States

## Abstract

Drug-induced changes in mitochondrial function have been described for many medications, including those commonly prescribed for older adults such as statins, NSAIDs, and metformin; however, the clinical impact of “mito-modulatory medication” use remains unclear. Given the high rate of prescription medication use among older adults, it is imperative to understand the consequences of mito-modulatory medication use. Further, mitochondrial dysfunction in skeletal muscle has been linked to declining physical performance and fitness in older adults, highlighting the importance of understanding how drug-induced mitochondrial modulation impacts skeletal muscle. We sought to assess how mito-modulatory medication use is related to skeletal muscle bioenergetics in older adults using data from the Study of Muscle, Mobility and Aging (SOMMA). We hypothesized that mito-modulatory medication use would be associated with lower skeletal muscle ex vivo (MAX ETS) and in vivo (ATPMax) bioenergetic measures. In unadjusted models, MAX ETS was lower in mito-modulatory medication users (mean= 82.4 [77.7-87.0]) compared to non-users (mean= 92.7 [87.5-97.9]) for men (p=0.0021) but not for women (p>0.05). Similarly, ATP-Max was lower in mito-modulatory medication users (mean= 0.54 [0.50-0.57]) compared to non-users (mean= 0.62 [0.58-0.66]) for men (p=0.0004). Adjustments for potential confounders attenuated some, but not all, of the associations. These results suggest that mito-modulatory medication use is related to lower skeletal muscle bioenergetics in older men but not women. Further investigation will provide greater clarity for the apparent sex differences in the impact of mito-modulatory medication use on muscle bioenergetics.

